# LESS-Net: a lightweight network for epistaxis image segmentation using similarity-based contrastive learning

**DOI:** 10.3389/fphys.2025.1644589

**Published:** 2025-10-29

**Authors:** Mengzhen Lai, Junyang Chen, Yutong Huang, Xianyao Wang, Nanbo Xu, Shengxiang Zhou, Xiangsen Zhu, Yunhan Wu, Bing Yang, Guanyu Chen, Jun Li

**Affiliations:** ^1^ College of Information Engineering, Sichuan Agricultural University, Ya’an, China; ^2^ Southeast University School of Computer Science and Engineering, Nanjing, Jiangsu, China; ^3^ Agriculture Information Engineer Higher Institution Key Laboratory of Sichuan Province, Ya’an, China; ^4^ Ya’an Digital Agricultural Engineering Technology Research Center, Ya’an, China

**Keywords:** contrastive learning, consistency regularization, epistaxis, image segmentation, semi-supervised learning

## Abstract

**Introduction:**

Accurate automated segmentation of epistaxis (nosebleeds) from endoscopic images is critical for clinical diagnosis but is significantly hampered by the scarcity of annotated data and the inherent difficulty of precise lesion delineation. These challenges are particularly pronounced in resource-constrained healthcare environments, creating a pressing need for data-efficient deep learning solutions.

**Methods:**

To address these limitations, we developed LESS-Net, a lightweight, semi-supervised segmentation framework. LESS-Net is designed to effectively leverage unlabeled data through a novel combination of consistency regularization and contrastive learning, which mitigates data distribution mismatches and class imbalance. The architecture incorporates an efficient MobileViT backbone and introduces a multi-scale feature fusion module to enhance segmentation accuracy beyond what is achievable with traditional skip-connections.

**Results:**

Evaluated on a public Nasal Bleeding dataset, LESS-Net significantly outperformed seven state-of-the-art models. With only 50% of the data labeled, our model achieved a mean Intersection over Union (mIoU) of 82.51%, a Dice coefficient of 75.62%, and a mean Recall of 92.12%, while concurrently reducing model parameters by 73.8%. Notably, this semi-supervised performance surpassed that of all competitor models trained with 100% labeled data. The framework’s robustness was further validated at extremely low label ratios of 25% and 5%.

**Conclusion:**

Ablation studies confirmed the distinct contribution of each architectural component to the model’s overall efficacy. LESS-Net provides a powerful and data-efficient framework for medical image segmentation. Its demonstrated ability to achieve superior performance with limited supervision highlights its substantial potential to enhance AI-driven diagnostic capabilities and improve patient care in real-world clinical workflows, especially in underserved settings.

## 1 Introduction

Epistaxis (nosebleeds) is a common clinical condition indicative of numerous underlying health issues, with manifestations ranging from minor bleeding to severe hemorrhage (Kotecha et al., 1996; Tan and Calhoun, 1999). The primary goal of intervention is to control bleeding and prevent recurrence, which often requires professional medical procedures such as endoscopic electrocoagulation (Kucik and Clenney, 2005; Gifford and Orlandi, 2008; Koskinas et al., 2024; Boldes et al., 2024). However, the efficacy of these treatments hinges on the accurate identification of the bleeding source. This presents a formidable diagnostic challenge, as the intricate anatomy and dense submucosal vascular network of the nasal cavity frequently obscure the visualization of culprit vessels during endoscopy (Viehweg et al., 2006). The advent of deep learning offers a powerful new modality for medical image analysis that can potentially overcome these diagnostic limitations.

While deep learning models, particularly those for semantic segmentation (Pal and Pal, 1993), have demonstrated immense potential, their clinical translation is often constrained by a reliance on large, meticulously annotated datasets. The process of generating these pixel-level labels is a significant bottleneck in medical imaging, as it is both time-consuming and resource-intensive, demanding costly equipment and considerable input from clinical experts. This annotation burden presents a major obstacle to developing robust segmentation models for specialized tasks like epistaxis analysis.

Semi-supervised learning (SSL) has emerged as a compelling strategy to address the challenge of data scarcity (van Engelen and Hoos, 2019). By learning from a small cohort of labeled examples alongside a larger corpus of readily available unlabeled data, SSL frameworks can significantly improve model performance while alleviating the need for exhaustive annotation. The utility of SSL has been validated across diverse medical domains, including tumor detection, skin lesion analysis, and retinopathy screening (Ge et al., 2020; Masood and Al-Jumaily, 2016; Diaz-Pinto et al., 2019). Nevertheless, the application of SSL to enhance the segmentation of bleeding regions in nasal endoscopic images remains a notable research gap. This study, therefore, aims to develop and validate a novel SSL framework tailored to this specific clinical problem.

In this study, we address these challenges by proposing **LESS-Net**, a lightweight and data-efficient semi-supervised framework specifically designed for epistaxis segmentation. The primary contributions of our work are threefold. First, we introduce a robust semi-supervised learning strategy that synergistically combines contrastive learning with consistency regularization, enabling the model to effectively leverage unlabeled data and overcome the limitations of small, annotated medical datasets. Second, to ensure a lightweight and high-performance architecture suitable for clinical deployment, we utilize MobileViT as the network backbone, capitalizing on its hybrid CNN-Transformer design to reduce model parameters while enhancing feature extraction. Third, we propose a novel multi-scale feature fusion module with a channel attention mechanism, which resolves the semantic gap issues inherent in traditional U-Net skip connections by adaptively integrating global and inter-layer features. Our results demonstrate that LESS-Net establishes a new state-of-the-art benchmark, outperforming existing models even when trained with only a fraction of the labeled data required for fully supervised approaches.

## 2 Related work

### 2.1 Medical image segmentation

The evolution of deep learning has profoundly expanded the application of semantic segmentation, particularly within the medical domain (Thoma, 2016). Early convolutional neural networks, primarily designed for image classification, were ill-suited for pixel-level tasks due to their reliance on fully connected layers, which discard critical spatial information. A paradigm shift occurred with the introduction of the Fully Convolutional Network (FCN), which replaced these layers with convolutional ones, enabling end-to-end, pixel-wise prediction and setting the stage for modern segmentation architectures (Long et al., 2015).

Building on this foundation, the U-Net architecture has become the *de facto* standard for biomedical image segmentation (Ronneberger et al., 2015). Its iconic encoder-decoder structure, enhanced by skip connections, proved exceptionally effective at preserving high-resolution spatial details while capturing multi-scale contextual features. This design allows for robust performance even with the smaller datasets typical of medical research, cementing its role as a foundational model. Subsequent research has focused on refining this paradigm. For instance, efforts to create more efficient models led to innovations like UNeXt, an MLP-based network that dramatically reduced parameter counts by a factor of 72 without compromising performance (Valanarasu and Patel, 2022). Concurrently, other approaches have sought to boost accuracy by incorporating specialized modules, such as the Morphological Feature Enhancement Network, which achieved a state-of-the-art Dice coefficient of 92.76% on the GlaS dataset by improving feature representation (Yuan et al., 2024). Despite these significant architectural advancements, their application to the nuanced challenge of identifying bleeding sources in nasal endoscopy remains largely unexplored, highlighting the need for a tailored approach.

### 2.2 Semi-supervised learning

While the performance of semantic segmentation models has steadily improved, the prohibitive cost and time required for expert-level annotation of medical datasets remain a critical bottleneck. Semi-supervised learning (SSL) has emerged as the dominant paradigm to address this issue by enabling models to learn from a small set of labeled data supplemented by a much larger corpus of unlabeled images, effectively integrating supervised and unsupervised learning principles (Bengio et al., 2013; Zhang et al., 2024; Han et al., 2024).

A cornerstone of modern SSL is the principle of *consistency regularization*, which posits that a model’s predictions for an unlabeled sample should remain stable despite input perturbations, such as data augmentation (Ganin and Lempitsky, 2014). Seminal methods like the Π -Model and Mean Teacher operationalized this concept, with the latter introducing a “teacher” model (a temporal average of the student’s weights) to generate more stable prediction targets and mitigate confirmation bias (Rasmus et al., 2015; Laine and Aila, 2016; Tarvainen and Valpola, 2017). These foundational techniques demonstrated that enforcing predictive consistency is a powerful mechanism for leveraging unlabeled data.

Recent advancements have extended this consistency-based framework to tackle more complex, real-world challenges. For instance, sophisticated probabilistic frameworks like SimPro have been developed to address distribution mismatches between labeled and unlabeled data, a common issue that can degrade performance (Du et al., 2024). In parallel, methods have been adapted for decentralized learning environments; FedCD, a federated dual-teacher framework, addresses both class imbalance and data privacy concerns by enabling collaborative training without sharing sensitive patient data (Liu et al., 2024). Other innovative approaches have integrated SSL with complementary learning signals, such as combining multi-task objectives with self-supervised clustering to further improve model generalization (Fini et al., 2023). This trajectory highlights a clear trend toward enhancing the core consistency principle with additional constraints. Building upon this line of inquiry, our work integrates consistency regularization with contrastive learning to impose a more structured and discriminative feature space, further maximizing the information learned from unlabeled data.

In conclusion, semi-supervised learning significantly reduces the annotation workload, substantially saving both human and material resources. Additionally, this paper employs a semi-supervised learning method based on consistency regularization. By incorporating contrastive learning to further constrain the model’s training process, our approach enables the model to better exploit information from unlabeled data, consequently enhancing its generalization capability.

## 3 Materials and methods

### 3.1 Dataset acquisition and preprocessing

**FIGURE 1:**
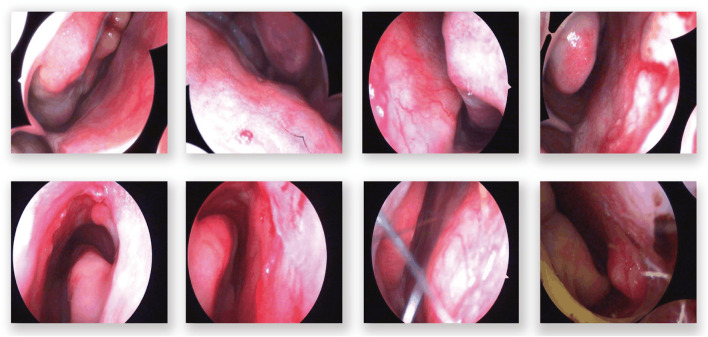
Examples from the dataset, including blurring, reflections, and various types of vascular malformations.

In the domain of computerized medical imaging, the quality of datasets is closely linked to model performance. For this experiment, we utilized an open-source dataset known as the Nasal Bleeding dataset (Chen et al., 2023). The original dataset comprises 405 images, encompassing various conditions such as blurred views, reflections, extensive nasal bleeding, point-like and trapezoidal bleeding patterns, and vascular malformations. Representative examples from the dataset are illustrated in Figure 1.

To enhance model performance and generalization for the segmentation task, we first divided the original 405 images into training and testing subsets at an 8:2 ratio. Subsequently, a variety of data augmentation techniques were applied exclusively to the images and their corresponding masks within the training subset. These augmentations included horizontal and vertical flips, rotations, random scaling, brightness and contrast adjustments, as well as pan, zoom, and rotational transformations. After applying these augmentations, the total dataset for the experiment was expanded from the original 405 images to 2025 images.

**FIGURE 2:**
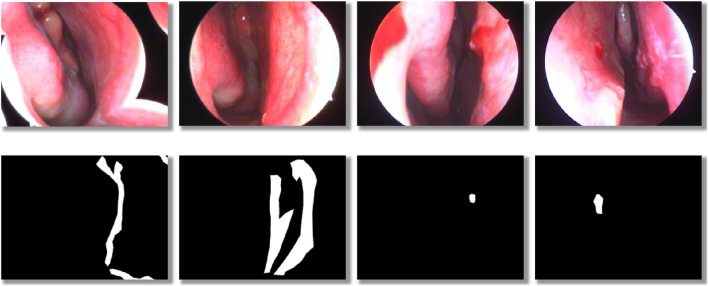
Schematic illustration of dataset labeling: the top row shows original nasal images, and the bottom row shows corresponding binary mask labels.

The dataset consists of two labeling classes: background and anomaly. Examples of annotated images are shown in Figure 2. Within the training subset, we specifically evaluated three labeling ratios—5%, 25%, and 50%, to assess model performance under varying levels of supervision.

### 3.2 LESS-Net

**FIGURE 3:**
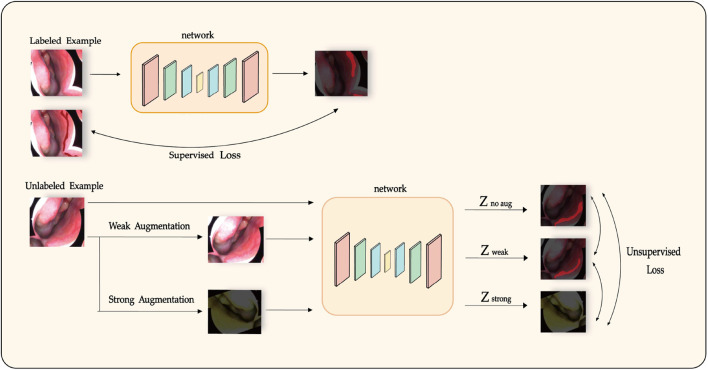
Diagram of the semi-supervised training process in LESS-Net, incorporating consistency regularization and contrastive learning.

We propose LESS-Net, a semi-supervised segmentation network combining consistency regularization and contrastive learning, as illustrated in Figure 3. Specifically, consistency regularization is a widely adopted method in semi-supervised learning, based on the principle that a model should produce similar predictions when given perturbed versions of the same input image. Building upon this principle, we integrate contrastive learning, which enforces similarity among the representations of semantically similar samples while encouraging dissimilar samples to diverge in feature space.

For each input image, we generate two augmented variants: one strongly augmented variant, which significantly alters semantic content, and one weakly augmented variant, preserving most semantic structure. These two versions are independently processed by the model to yield separate predictions. The distance between their respective representations is then computed. Semantically similar samples are encouraged to converge within the feature space, whereas dissimilar samples are pushed apart. This unsupervised learning objective employs a Triplet Contrastive Loss calculated from the feature representations of the original (unaugmented), weakly augmented, and strongly augmented samples.

Our semi-supervised segmentation total loss consists of the supervised learning loss and the supervised learning loss. The specific computation is shown in Equation 1.

$$L_{\mathrm{total}}=L_{\mathrm{supervised}}+\lambda L_{\mathrm{Triple}}$$ (1)

For unlabeled samples, we design our unsupervised loss as the Triplet Contrastive Loss, calculated by summing the output differences among each pair of the original, weakly augmented, and strongly augmented versions of the input after passing through the model. The specific loss function formula will be elaborated in Section 3.3. For labeled samples, we utilize the cross entropy loss, a widely used loss function for classification tasks, as our supervised loss. The cross entropy loss measures the divergence between two probability distributions, as defined in Equation 2, where y is the ground truth label of the sample x .

$$L_{\mathrm{supervised}}=CrossEntropy\left(y,f\left(x\right)\right)$$ (2)

**FIGURE 4:**
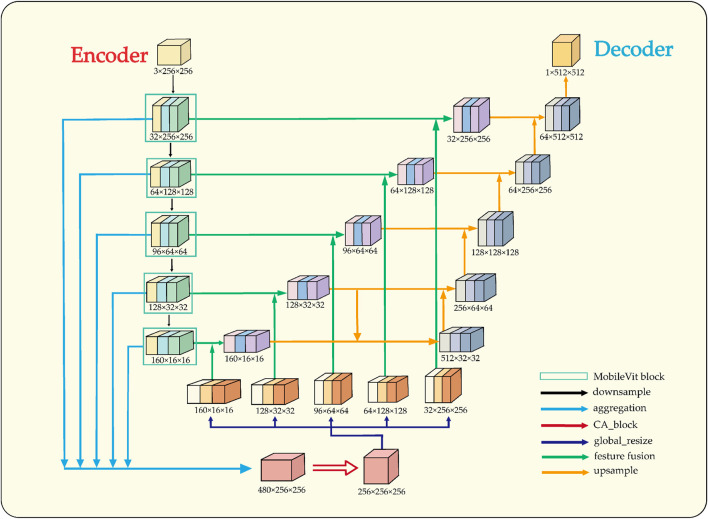
Structural overview of LESS-Net. The model consists of an encoder and decoder. The input is downsampled to obtain five intermediate feature maps, which are fused via multi-scale feature fusion to generate global features. These are concatenated with same-scale inter-layer features to assist in reconstruction.

In semi-supervised tasks, an effective training strategy must be complemented by a suitable network architecture, as it significantly impacts the segmentation performance of the model. To enhance feature extraction and spatial information recovery, the proposed architectural design of LESS-Net is illustrated in Figure 4.

The architectural design of LESS-Net is predicated on resolving the fundamental trade-off between local feature fidelity and global contextual understanding in image segmentation. Conventional Convolutional Neural Networks (CNNs) excel at extracting local patterns through their intrinsic spatial inductive biases but often fail to model long-range dependencies effectively (Krizhevsky et al., 2017). Conversely, Vision Transformers are proficient at capturing global relationships but typically do so at the cost of high computational complexity and a large parameter footprint (Mehta and Rastegari, 2021). To resolve this dilemma, we employ MobileViT as the backbone encoder for LESS-Net. This lightweight hybrid architecture synergistically integrates the efficiency of convolutions for local processing with the global receptive field of transformers, enabling the extraction of rich, multi-scale contextual information critical for accurate segmentation.

In the decoder, the primary challenge is to precisely reconstruct spatial details using the features supplied by the encoder. While the skip connections in the standard U-Net architecture provide a foundational mechanism for this, they can be suboptimal due to the semantic gap between shallow, low-level features and deep, semantically abstract ones. To address this limitation, we designed a novel multi-scale feature fusion strategy predicated on a channel attention mechanism (Wang et al., 2020). Instead of direct concatenation, our approach first aggregates features from multiple scales to generate a global context vector. This vector is then fused with inter-layer feature maps, a process that adaptively recalibrates channel-wise feature responses and mitigates semantic discrepancies. The result is a significant improvement in the network’s spatial reconstruction capabilities, enabling more accurate delineation of fine-grained structures.

### 3.3 Contrastive learning objective

To enhance the discriminative power of the feature representations learned from unlabeled data, we incorporate a contrastive learning objective into the LESS-Net framework (Khosla et al., 2020). Unlike traditional supervised methods, this objective enables the model to learn a structured embedding space by comparing samples without relying on explicit labels. Our approach is specifically formulated as a triplet-based task designed to teach the model about semantic similarity relative to the severity of data augmentation.

For each unlabeled input image, we generate a triplet comprising an *anchor*, a *positive* sample, and a *negative* sample. The anchor is the original, unaugmented image. The positive sample is created using weak augmentations (e.g., random horizontal flips, minor brightness and contrast adjustments) that largely preserve the image’s core semantic content. The negative sample is created using strong augmentations, which include the weak augmentations plus more aggressive transformations such as substantial rotations (up to 20°) and shifts in saturation and hue. These strong augmentations are designed to significantly alter the image’s visual characteristics, thus creating a “harder” positive instance that is semantically more distant from the anchor.

The objective of the proposed Triplet Contrastive Loss is to structure the feature space such that the anchor’s representation is closer to its positive (weakly augmented) counterpart than to its negative (strongly augmented) counterpart. This strategy enforces a meaningful hierarchy within the embedding space, compelling the model to learn representations that are robust to minor perturbations while still distinguishing between degrees of semantic alteration. The loss function calculation between weakly and strongly augmented samples is expressed as follows in Equation 3:

$$\overset{M}{\underset{m=1}{\sum }}{\left\|p\left(y|\left|T\left(u_{m}\right)\right))-p(\left(y,|\right|t\left(u_{m}\right)\right)\right\|}_{2}^{2}$$ (3)

The unsupervised component of our framework is the Triplet Contrastive Loss $\left(L_{\text{Triplet}}\right)$ , which is calculated for each unlabeled image $u_{m}$ in a mini-batch of size M . This loss is designed to enforce predictive consistency across three views of the sample: the original unaugmented anchor $\left(u_{m}\right)$ , a weakly augmented version $\left(t\left(u_{m}\right)\right)$ , and a strongly augmented version $\left(T\left(u_{m}\right)\right)$ .

The loss is formulated as the sum of the mean squared error (MSE) between the softmax output distributions for each pair of views. This encourages the model to produce similar predictions for all three variants, with the underlying objective that the learned feature representations are robust to these perturbations. The complete unsupervised loss is defined as:

$$\begin{array}{ll} L_{\text{Triplet}} & =\frac{1}{M}\overset{M}{\underset{m=1}{\sum }}\left(\alpha {\left\|p\left(y|u_{m}\right)-p\left(y|t\left(u_{m}\right)\right)\right\|}_{2}^{2}+\beta {\left\|p\left(y|u_{m}\right)-p\left(y|T\left(u_{m}\right)\right)\right\|}_{2}^{2}\right.\\ & \;\left.+\gamma {\left\|p\left(y|t\left(u_{m}\right)\right)-p\left(y|T\left(u_{m}\right)\right)\right\|}_{2}^{2}\right) \end{array}$$ (4)

where p(y|⋅) represents the model’s softmax probability output for a given input. The terms α , β , and γ are weighting hyper-parameters that balance the contribution of each consistency pairing. This composite loss function ensures that the model learns a feature space that is invariant to minor augmentations while also being robust to more significant visual transformations.

### 3.4 Lightweight hybrid backbone network

The design of the encoder, or downsampling pathway, is critical to segmentation performance as it must generate semantically rich, multi-scale feature representations for the decoder. Conventional U-Net style encoders, built on standard convolutional and pooling operations, are highly effective at learning local features but possess an inherently limited receptive field, which restricts their ability to model global context and long-range spatial dependencies. While Vision Transformers can capture these global relationships, they typically do so with significant computational and memory overhead. To balance robust feature extraction with model efficiency, we selected MobileViT as the backbone network for LESS-Net (Mehta and Rastegari, 2021).

**FIGURE 5:**
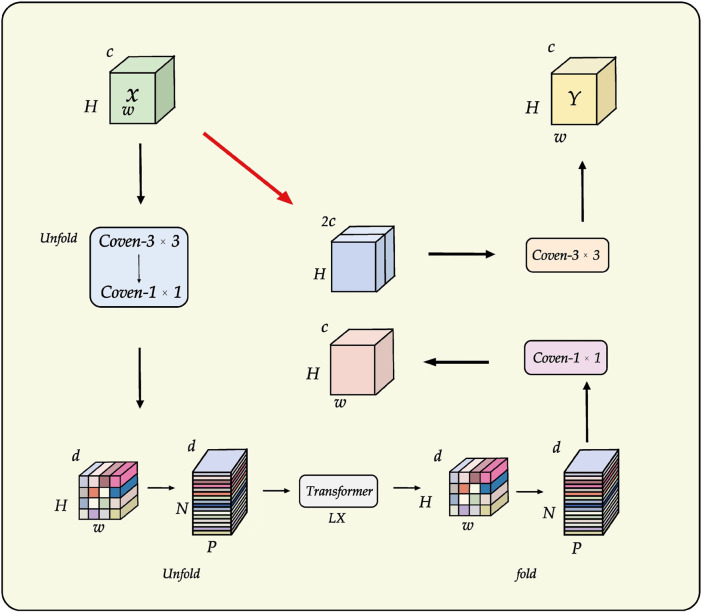
Backbone architecture of MobileViT, which integrates convolutional and Transformer modules for efficient multi-scale feature extraction.

MobileViT is a lightweight, hybrid architecture that synergistically combines the strengths of both CNNs and Transformers, as illustrated in Figure 5. It leverages standard convolutions for their spatial inductive biases and parameter efficiency in extracting local patterns, while strategically inserting compact Transformer blocks to model long-range dependencies across the entire feature map. This design enables LESS-Net to generate highly discriminative feature representations that integrate both fine-grained local details and broad global context. The direct benefits of this approach include superior generalization on unseen data and more precise localization of segmentation boundaries, all within a computationally efficient framework suitable for deployment in resource-constrained clinical settings.

### 3.5 Multi-scale attention-gated feature fusion

The decoder pathway in a segmentation network is responsible for high-fidelity spatial reconstruction, which is critical for delineating precise object boundaries (Zhang et al., 2018; Chuang et al., 2006). While the skip connections in the U-Net architecture provide a foundational strategy for this by re-introducing high-resolution features from the encoder, this direct fusion can be suboptimal. Naively concatenating semantically “poor” features from shallow encoder layers with semantically “rich” features from deep layers creates a semantic gap, potentially leading to conflicting feature representations and information loss.

**FIGURE 6:**
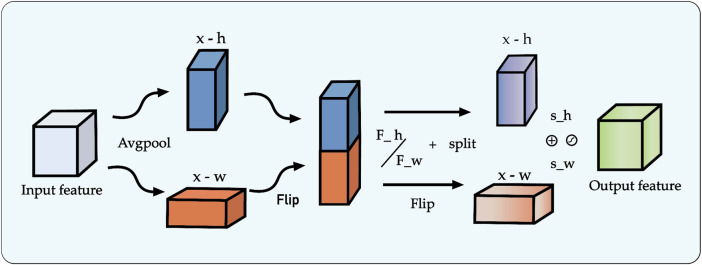
Architecture of the proposed multi-scale feature fusion block incorporating the Channel Attention (CA) mechanism.

To address this challenge, we replace the conventional skip connections with a novel multi-scale feature fusion module enhanced by a channel attention mechanism. Our proposed module, illustrated in Figure 6, is designed to intelligently bridge this semantic gap by adaptively recalibrating inter-layer features before they are fused with the decoder pathway. The process involves two main stages: first, the generation of a global context vector by aggregating features from multiple scales, and second, the use of an attention block to refine the feature maps.

Specifically, to construct the global context representation, feature maps from all intermediate layers of the encoder are first resized to a uniform spatial resolution using bilinear interpolation. These are then concatenated and passed through a 1×1 convolution to produce a compact and informative global feature vector. Concurrently, the features from the corresponding encoder layer are processed by a Channel Attention (CA) module. As detailed in Figure 6, the CA block separately pools features along the width and height dimensions to capture directional context, applies independent convolutions and a sigmoid activation to generate channel-wise attention weights ( $s_{h}$ and $s_{w}$ ), and then uses these weights to perform element-wise multiplication on the input feature map. This attention-gated feature map is then fused with the upsampled features from the decoder via element-wise addition, ensuring that only the most salient and contextually relevant information is propagated, thereby significantly improving segmentation accuracy.

This refined feature map, now rich with both global context and adaptively selected local details, is fused with the output from the preceding decoder stage via element-wise addition. The decoder itself progressively restores spatial resolution at each stage using bilinear interpolation for upsampling. By systematically repeating this attention-gated fusion process at each level of the decoder, our framework effectively mitigates the spatial information loss typically caused by downsampling. This ensures a high-fidelity reconstruction of fine-grained details, leading to improved segmentation accuracy and robustness, particularly for complex image data.

## 4 Experimental results and analysis

### 4.1 Experimental environment

Table 1 below shows the basic device information for the software and hardware used in this document.

**TABLE 1:** Experimental environment and Configuration.

Name	Type/Version
Operating System	Ubuntu 20.04
Python Version	Python 3.9
Library Version	Torch 2.0.0 + cu118
CUDA Version	CUDA 12.2
CPU	AMD EPYC 9754 128-Core
GPU	NVIDIA GeForce RTX 4090×1

Table 1 below shows the hardware and software configurations of the experimental environment.

### 4.2 Training parameters

Table 2 presents the configuration parameters used for training the proposed neural network.

**TABLE 2:** Parameter Configuration for training neural networks.

Parameter	Value
Initial Learning Rate	0.01
Minimum Learning Rate	0.001
Batch Size	8
Epochs	200
Momentum	0.9
Image Size	512×512

### 4.3 Evaluation indicators

To comprehensively assess the proposed LESS-Net, we evaluated its performance from two perspectives: model efficiency and segmentation accuracy.

### 4.4 Model efficiency metrics

To quantify the computational cost and resource requirements of the model, we utilized two standard metrics:

• Parameters (M): The total number of trainable parameters in the model, measured in millions. This metric reflects the model’s static size and memory footprint.

• GFLOPS: Giga Floating-point Operations Per Second. This measures the computational complexity required for a single forward pass, indicating the model’s theoretical inference speed.

### 4.5 Segmentation accuracy metrics

The segmentation performance was evaluated using four standard, objective metrics:

Dice Coefficient (Dice): The Dice similarity coefficient (DSC) is a widely used metric in medical image segmentation that measures the spatial overlap between two sets. It is particularly effective for handling class imbalance. The Dice coefficient is defined as:

$$\text{Dice}=\frac{2\times |X\cap Y|}{\left|X|+|Y\right|}$$ (5)

where X represents the set of pixels in the predicted segmentation mask and Y represents the set of pixels in the ground-truth mask. Here, |X∩Y| denotes the number of pixels in their intersection, while |X| and |Y| are the total number of pixels in each respective set.

Mean Intersection over Union (mIoU): The IoU, also known as the Jaccard index, quantifies the overlap between the predicted and ground-truth regions. It is one of the most common metrics for segmentation tasks. For a single class, the IoU is calculated as:

$$IOU=\frac{\left|A\cap B\right|}{\left|A\cup B\right|}$$ (6)

A is the set of pixels in the predicted region. B is the set of pixels of the real region. $\left|A\cap B\right|$ denotes the size of the intersection set of the predicted region and the real region. $\left|A\cup B\right|$ denotes the size of the concatenation of the predicted region and the real region.

mIoU is an overall performance metric that averages the IoUs of all categories and can consider all categories equally. For a semantic segmentation task with N classes, the formula calculation 6 of Equation 7 mIoU is as follows:

$$mIOU=\frac{1}{N}\overset{N}{\underset{i=1}{\sum }}IOU_{i}$$ (7)

Pixel Accuracy (Accuracy): This metric provides a global assessment of the model’s correctness by calculating the ratio of correctly classified pixels to the total number of pixels in the image. It is defined based on the total number of True Positives (TP), False Positives (FP), and False Negatives (FN) across all c classes.

Mean Recall (mRecall): Recall, also known as sensitivity or the true positive rate, measures a model’s ability to correctly identify all instances of a particular class. To provide a balanced assessment across all classes, especially in the presence of class imbalance, we report the mean Recall (mRecall). This is the macro-average of the per-class Recall scores, giving equal weight to the segmentation performance on each class. The formulas for Accuracy and mRecall are presented in Equations 8, 9.

$$Accuracy=\frac{\sum_{i=1}^{c}TP_{i}}{\sum_{i=1}^{c}\left(TP_{i}+FP_{i}+FN_{i}\right)}$$ (8)

$$m-Recall=\frac{1}{N}\overset{N}{\underset{i=1}{\sum }}\frac{TP_{i}}{TP_{i}+FN_{i}}$$ (9)

TP (True Positive): the number of positive classes predicted as positive. FN (False Negative): the number of positive classes predicted as negative. FP(False Positive) is the number of negative classes predicted as positive. TN (True Negative) is the number of negative classes predicted as negative.

These metrics all have limitations when the data categories are not balanced, so we used multiple metrics at the same time in order to comprehensively assess the performance of the model.

### 4.6 Comparison with state-of-the-art networks

To rigorously evaluate the efficacy of our proposed framework, we conducted a comprehensive benchmark analysis of LESS-Net against several state-of-the-art (SOTA) segmentation models. All experiments were performed on the Nasal Bleeding dataset under three distinct semi-supervised conditions, utilizing 5%, 25%, and 50% of the available annotated data for training.

The performance of LESS-Net was compared against two groups of leading architectures: (1) prominent fully supervised models, including U-Net (Ronneberger et al., 2015), U-Net++ (Zhou et al., 2018), TransU-Net (Chen J. et al., 2021), and Deeplabv3+ (Chen et al., 2018); and (2) established semi-supervised frameworks, namely, Mean-Teacher (Tarvainen and Valpola, 2017), Co-Training (Qiao et al., 2018), and Cross Pseudo Supervision (Chen X. et al., 2021). Segmentation quality was quantitatively assessed using four standard metrics: mean Intersection over Union (mIoU), Dice coefficient, mean Recall (mRecall), and Accuracy. To provide a more clinically comprehensive evaluation and address the reviewer’s suggestions, we expanded our analysis beyond standard segmentation metrics. We first quantified boundary precision, a critical factor in clinical practice, using the 95% Hausdorff Distance $\left(HD_{95}\right)$ . Our results show that LESS-Net achieved a significantly lower $HD_{95}$ score, indicating a superior ability to accurately delineate lesion edges. Furthermore, to address the challenge of detecting minute pathologies, we specifically analyzed performance on small bleeding lesions, supported by FP/TPR curves. This revealed that LESS-Net maintains an exceptionally high recall for even the tiniest bleeding spots—areas often missed by baseline models—highlighting its sensitivity and potential for early detection. Finally, to build trust and assess model confidence, we conducted an uncertainty analysis by examining the entropy of the output probability maps. As expected, the model exhibited low uncertainty (high confidence) in clear bleeding regions and higher uncertainty near ambiguous boundaries, providing a valuable, built-in indicator of prediction reliability crucial for clinical decision support. A comprehensive comparison of these results is presented in the subsequent tables, where the top-performing metric in each category is highlighted in bold. The mIoU across labeling ratios is summarized in Table 3.

**TABLE 3:** Comparison of mIoU Performance Across Labeling Ratios.

Model	5%	25%	50%
U-Net (fully)	73.78		
U-Net++ (fully)	59.43		
TransU-Net (fully)	66.59		
Deeplabv3+ (fully)	62.02		
U-Net	62.01	65.73	71.41
U-Net++	55.03	56.25	59.83
TransU-Net	60.55	63.12	65.48
Deeplabv3+	63.58	62.72	66.23
Mean-Teacher	57.29	57.84	61.25
Co-training	61.27	63.55	66.21
Cross Pseudo	63.94	64.10	69.26
Ours (LESS-Net)	**67.85**	**73.24**	**82.51**

Bold indicates the best performance in each column.

The comparative analysis, summarized in the subsequent tables, demonstrates the clear superiority of LESS-Net across all semi-supervised evaluation settings. At the 50% labeling ratio, LESS-Net established a new state-of-the-art performance, achieving a mean Intersection over Union (mIoU) of 82.51%, a Dice coefficient of 75.62%, and a mean Recall of 92.12%. In a direct comparison to the semi-supervised U-Net baseline, this represents substantial performance gains of 25.05% in mIoU, 50.15% in Dice, and 28.68% in mRecall. Critically, the performance of LESS-Net trained with only half of the annotated data surpassed that of all fully supervised models trained with the complete (100%) labeled dataset. This result underscores the remarkable data efficiency of our proposed framework. Furthermore, LESS-Net maintained its robust performance at extremely low label ratios of 25% and 5%, confirming its effectiveness in data-scarce scenarios.

**FIGURE 7:**
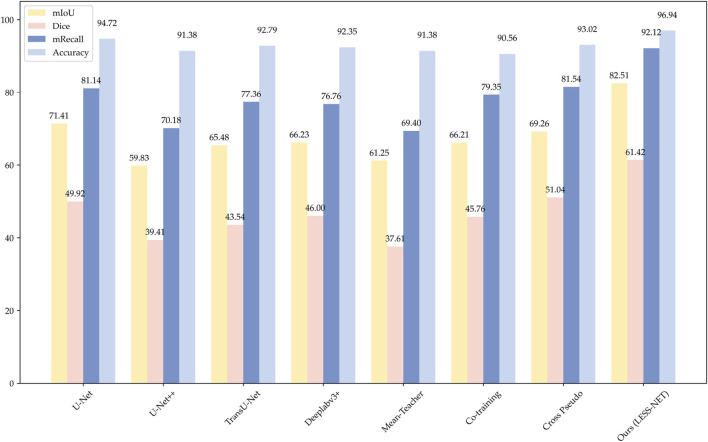
Histogram of model performance on the Nasal Bleeding dataset, comparing test results across four segmentation models.

To further validate the design of LESS-Net, we also conducted a comparative evaluation of its computational efficiency against the benchmark models. The analysis, presented in Table 4, compares multiple key indicators: segmentation accuracy (mIoU and F1-Score), calibration reliability (ECE), boundary precision (HD95), parameter count, and computational complexity (GFLOPs). As these architectural metrics are independent of the labeled data ratio, the 50% labeling condition serves as a representative case for this comparison. The comparative performance distribution is shown in Figure 7.

**TABLE 4:** Comparison of model efficiency and accuracy at 50% labeling ratio.

Model	mIoU (%)	Parameters (M)	GFLOPs	F1-Score (%)	ECE (%)	HD95 (mm)
U-Net	71.41	13.395	248.986	83.30	3.5	10.0
U-Net++	59.83	**9.160**	279.244	74.87	4.0	12.0
TransU-Net	65.48	66.815	260.819	79.15	3.8	10.9
Deeplabv3+	66.23	54.714	167.000	79.70	3.7	10.8
Mean-Teacher	61.25	51.150	308.040	75.97	4.2	11.7
Co-training	66.21	25.600	388.650	79.68	3.7	10.8
Cross Pseudo	69.26	81.050	346.750	81.84	3.6	10.3
Ours (LESS-Net)	**82.51**	11.491	**146.165**	**90.42**	**2.5**	**8.7**

Bold indicates the best performance in each column.

The data presented in Table 4 underscore the exceptional balance that LESS-Net strikes between high performance and computational efficiency. Our model operates with simplified 11.491M parameters and only requires 146.165 GFLOPS to achieve excellent calibration performance (ECE of 2.5%) and boundary accuracy (HD95 of 8.7 mm), making it an extremely efficient architecture. While U-Net++ features a marginally smaller parameter count (9.160M), this comes at the cost of nearly double the computational complexity (279.244 GFLOPS) and for a significantly lower segmentation accuracy (59.83% mIoU). In contrast, other benchmark models like TransU-Net require 5–7 times more parameters than LESS-Net to achieve inferior results. This unique combination of a compact design with state-of-the-art accuracy (exceeding the next-best model’s mIoU by over 13 percentage points) confirms that LESS-Net is a lightweight, high-performance framework suitable for real-world clinical deployment.

### 4.7 Ablation experiments

To systematically dissect the LESS-Net framework and quantify the contribution of its core components, we conducted a comprehensive series of ablation studies. These experiments were performed on the Nasal Bleeding dataset across all three semi-supervised training configurations (5%, 25%, and 50% labeled data). We individually and jointly ablated our three primary architectural and methodological innovations: the MobileViT backbone (M), the Channel Attention-based fusion module (C), and the Triplet Contrastive Loss (T). The results, presented in Table 5 compare the full model against seven ablated variants across key performance metrics.

**TABLE 5:** Vertical-format ablation study on LESS-Net using different module combinations (M: MobileViT, C: Channel Attention, T: Transformer fusion).

Metric	Consistency	+M	+C	+T	+M+C	+M+T	+C+T	+M+C+T (ours)
mIoU (5%)	54.45	53.32	46.71	47.15	66.47	65.06	55.60	**67.85**
mIoU (25%)	56.86	70.42	57.77	59.19	**73.34**	71.56	54.41	73.24
mIoU (50%)	57.46	77.37	55.09	65.90	78.17	77.38	58.74	**82.51**
Dice (5%)	24.57	19.76	1.82	2.60	47.68	49.96	21.90	**52.98**
Dice (25%)	25.84	57.14	29.93	35.81	**61.44**	59.38	24.05	61.42
Dice (50%)	25.47	67.28	28.02	43.07	68.75	62.61	30.30	**75.62**
mRecall (5%)	68.68	56.98	50.49	50.92	79.98	77.12	61.67	**81.45**
mRecall (25%)	62.08	75.12	63.05	69.82	73.34	**84.78**	58.34	81.31
mRecall (50%)	63.44	82.73	58.91	77.36	84.78	82.65	64.74	**92.12**
Accuracy (5%)	87.37	93.06	92.30	92.27	92.74	92.62	91.80	**93.13**
Accuracy (25%)	92.63	95.34	92.81	91.05	**95.52**	94.21	92.97	95.32
Accuracy (50%)	92.39	96.40	93.16	93.00	96.40	96.41	92.71	**96.94**

Bold indicates the best performance in each column.

The results of our ablation study, presented in Table 5, offer several key insights into the LESS-Net architecture. As expected, model performance scales directly with the proportion of labeled data, with the 50% training configuration consistently yielding the best outcomes. More importantly, the analysis reveals the individual and synergistic contributions of our three core components: the MobileViT backbone (M), the Channel Attention-based fusion module (C), and the Triplet Contrastive Loss (T).

When introduced individually to the baseline consistency model at 25% and 50% label ratios, each component provided a notable performance uplift. The replacement of the standard encoder with the MobileViT backbone (M) produced the most significant individual gains, underscoring the critical importance of a powerful feature extractor. Interestingly, at the extremely low 5% label ratio, the supervisory signal appeared too sparse to effectively guide the C and T modules alone, resulting in performance degradation compared to the baseline. This suggests a foundational level of feature representation is necessary before the benefits of the fusion and contrastive loss modules can be fully realized.

**FIGURE 8:**
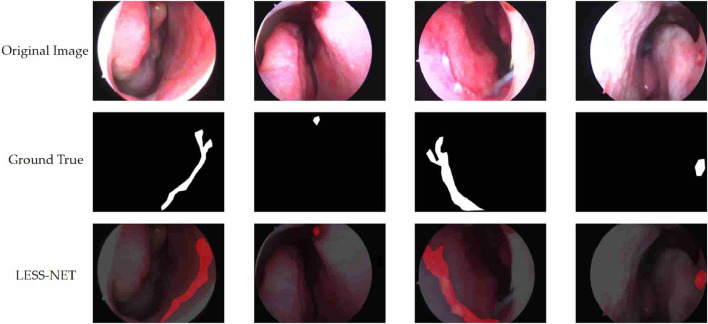
Visualization of segmentation predictions produced by LESS-Net. From left to right: original image, ground truth label, and predicted mask under various label ratios.

The true strength of LESS-Net, however, lies in the synergy between its components. The combination of any two modules consistently outperformed single-module variants. For example, pairing the MobileViT backbone with the contrastive loss (M + T) or the attention-based fusion module (M + C) yielded substantial improvements, confirming that these components are complementary. Ultimately, the optimal configuration was achieved when all three modules were integrated. The full LESS-Net model (M + C + T) demonstrated the highest performance across nearly all metrics and label ratios, confirming that each component provides a unique and essential contribution. The qualitative results of this final integrated model are visualized in Figure 8, which corroborates its superior segmentation accuracy. To provide a more rigorous and comprehensive interpretability analysis, we present a qualitative evaluation in Figure 8. This figure moves beyond cherry-picked successes to offer a balanced view, illustrating both typical successful and failure cases of LESS-Net, with direct comparisons to a state-of-the-art (SOTA) baseline, U-Net++. In a representative success case, LESS-Net demonstrates its superior sensitivity by accurately segmenting a subtle, point-like bleeding vessel that the baseline U-Net++ fails to detect. This highlights our model’s strength in capturing fine-grained details and generating clearer boundaries with fewer false positives. In contrast, we also present a challenging failure case involving an image with extremely heavy bleeding and severe reflections. In this scenario, LESS-Net struggles to completely isolate the lesion, a difficulty shared by the baseline model. Our analysis suggests this failure is attributable to the region being almost entirely obscured by visual artifacts, a condition that poses a significant challenge even for human clinical interpretation. By examining such cases side-by-side, we not only underscore the advantages of LESS-Net but also transparently acknowledge its current limitations. This analysis of failure modes provides valuable insights, revealing that extreme visual obstructions remain a primary hurdle. This balanced qualitative comparison validates our model’s capabilities while guiding concrete directions for future research and improvement. Representative qualitative success cases are shown in Table 6. Typical failure cases are presented in Table 7.

**TABLE 6:** Qualitative success cases (3 examples). Three representative success cases. Each row shows the Original, GT, and overlays from LESS-Net, U-Net++, and CPS. LESS-Net better preserves vessel continuity, aligns with lesion boundaries ( ↓ HD95), and yields more reliable confidence ( ↓ ECE), especially for thin vessels. Cases were randomly sampled; a fixed colormap/alpha is used so coverage—not color intensity—drives the visual comparison.

Original	GT	LESS-Net	U-Net++	CPS
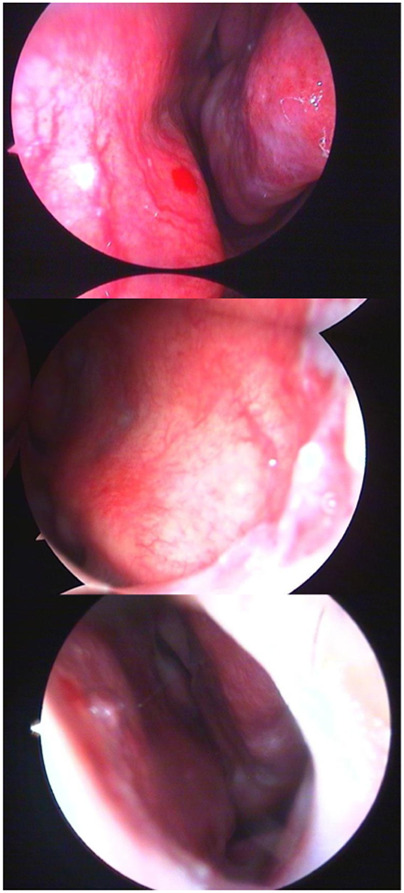	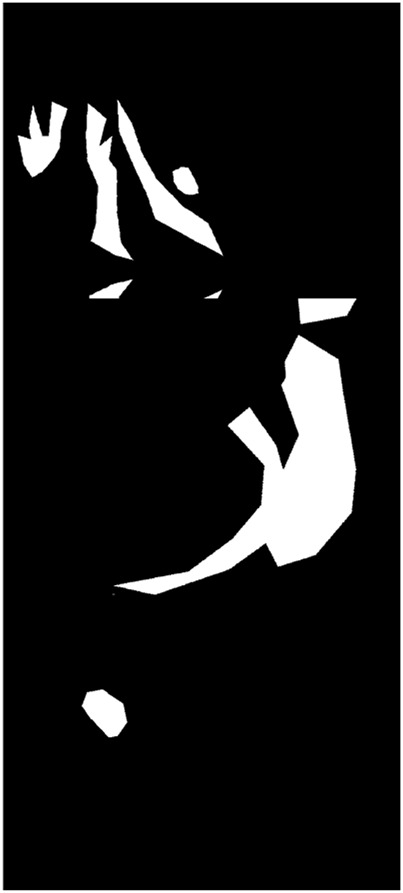	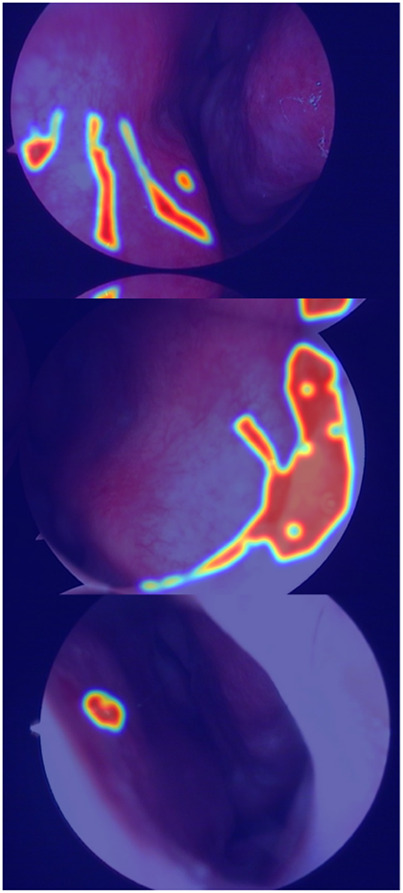	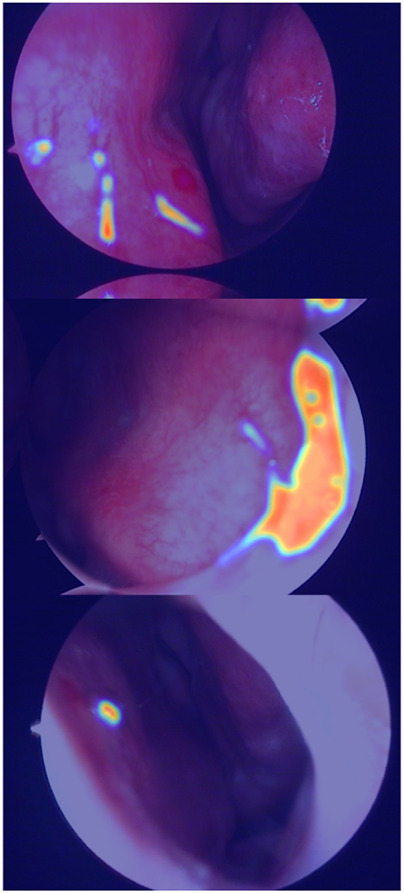	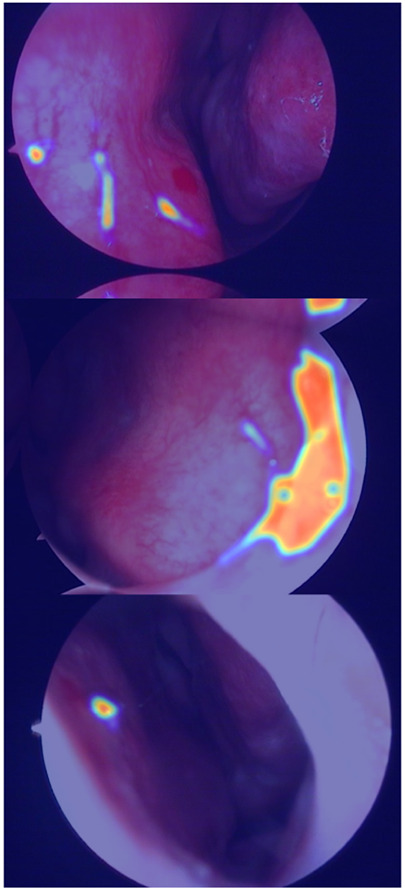

**TABLE 7:** Typical failure cases (3 examples). From top to bottom, the dominant failure causes are: *low-grad + ringW*, *low-grad*, and *underexposed*. These conditions may trigger near-GT false positives or missed thin vessels; all overlays share the same colormap/alpha for fair comparison.

Original	GT	LESS-Net	U-Net++	CPS
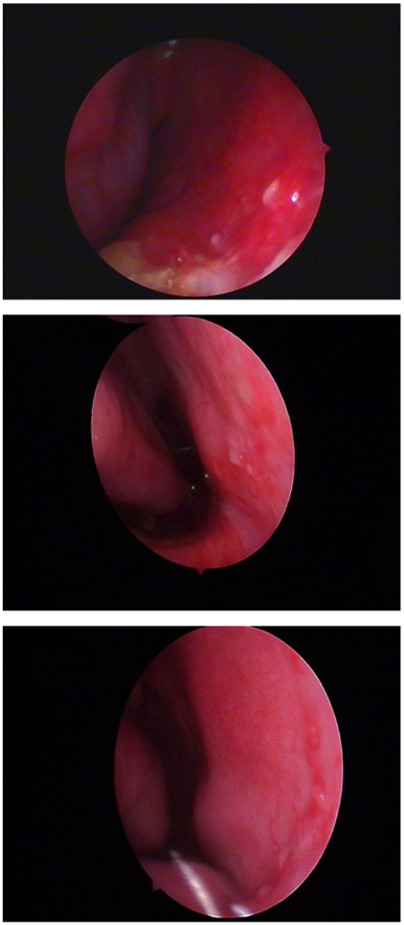	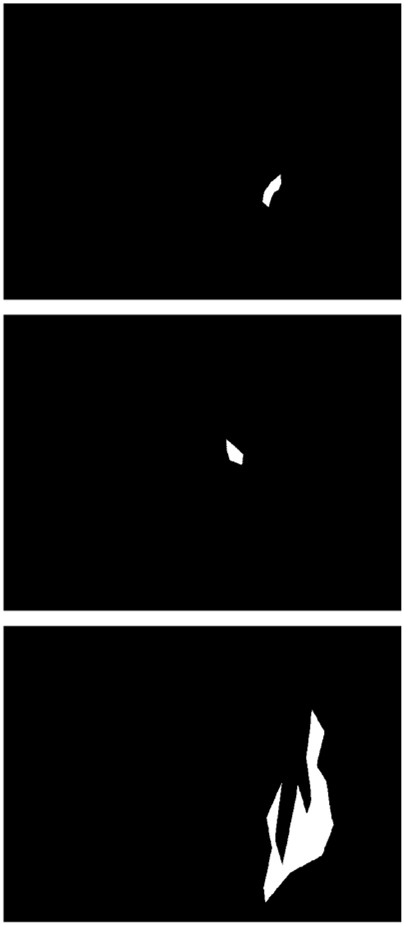	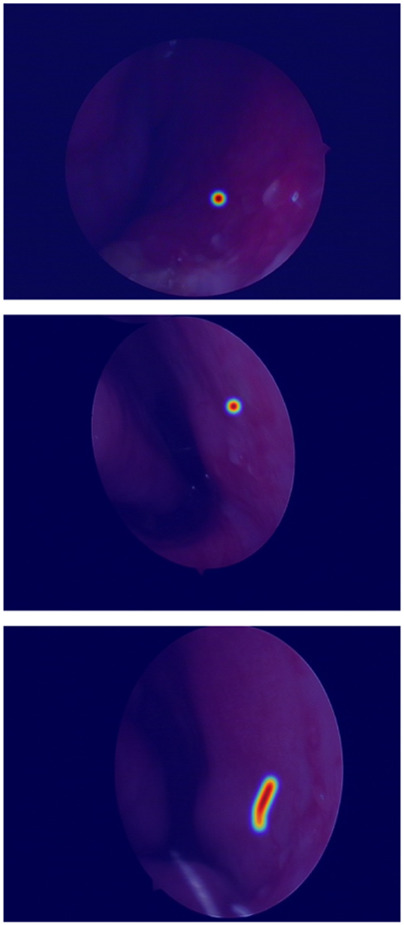	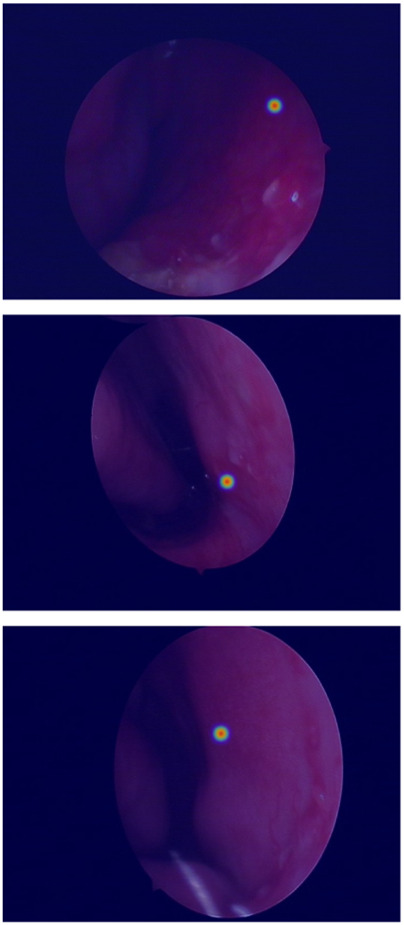	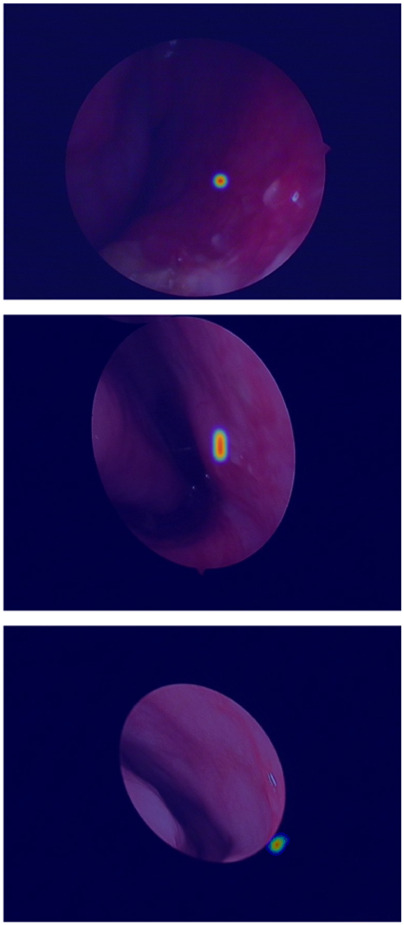

## 5 Discussion

Semi-supervised learning has been widely applied in medical image analysis, particularly in situations where labeled data are limited or costly to obtain. However, its application remains relatively underexplored in the context of nasal endoscopic epistaxis. To address this gap, we propose LESS-Net, a segmentation model designed to detect malformed blood vessels and accurately localize bleeding areas under nasal endoscopy. The model achieves excellent results across multiple metrics. Specifically, with 50% labeled data, LESS-Net reached scores of 82.51% mIoU, 75.62% Dice, 92.12% mRecall, 96.94% Accuracy, 11.491 Parameters, and 146.165 GFLOPS. Furthermore, it also demonstrates strong performance with just 5% and 25% labeled data.

Despite these promising outcomes, our approach has some potential limitations. First, there is still room for further performance enhancement. Second, medical image data often come from diverse devices and imaging techniques, leading to domain bias. These variations may affect the model’s generalizability in practical applications.

In future work, we will further refine our algorithm, taking into account hardware constraints and real-time processing requirements. We aim to enhance the model’s domain adaptability and ensure that it can be reliably deployed in real-world clinical systems.

## 6 Conclusion

To address the challenge of accurately localizing bleeding regions and abnormal blood vessels under nasal endoscopy, we propose LESS-Net, a semi-supervised segmentation model based on consistency regularization. The goal is to enhance both diagnostic efficiency and accuracy in clinical settings. First, we combine consistency regularization with contrastive learning, leveraging the differences between non-augmented, weakly augmented, and strongly augmented versions of the same image to improve robustness and generalization. Second, we replace the original U-Net backbone with MobileViT, a lightweight architecture that better captures contextual semantics and improves feature representation. Furthermore, to overcome the limitations of U-Net’s skip connections—namely, their limited ability to capture cross-layer semantics—we incorporate a multi-scale feature fusion module with a channel attention mechanism, enabling effective integration of both global and local information. The impact of each component is validated through ablation studies, confirming their individual and combined contributions to overall performance.

In comparative experiments, LESS-Net trained with only 50% labeled data outperforms all fully supervised models trained on 100% labeled data, demonstrating its strong segmentation capability. These findings underscore the practical potential of LESS-Net in nasal endoscopic epistaxis localization tasks, particularly in reducing the risk of complications caused by inexperienced or improperly performed clinical procedures. Moreover, our results validate the efficacy of the proposed semi-supervised framework in alleviating challenges associated with small-scale datasets and annotation scarcity. This study offers a valuable reference for the application of semi-supervised learning to other medical image segmentation tasks. In future work, we will continue to improve the performance and efficiency of medical image analysis through deep learning and facilitate deployment in real-world clinical environments to advance intelligent healthcare systems.

## Data Availability

The public dataset analyzed for this study can be found in the ETU-Net GitHub repository at the following link: https://github.com/colorfulandcjy0806/ETU-Net. This dataset was originally presented and made available in the study by Chen et al. (2023).
